# Reassessing Normal Voiding Standards: A Cross-Sectional Study Based on Medical Professionals’ Evaluations with Portable Uroflowmetry and IPSS

**DOI:** 10.3390/jcm13102857

**Published:** 2024-05-12

**Authors:** Furkan Almas, Muhammed Furkan Dasdelen, Zuleyha Seyhan, Maral Sargolzaeimoghaddam, Arya Sarg, Omer Unlu, Zehra Betul Dasdelen, Rahim Horuz, Selami Albayrak, Mehmet Kocak, Pilar Laguna, Jean de la Rosette

**Affiliations:** 1 International School of Medicine, Istanbul Medipol University, 34810 Istanbul, Türkiye; m.f.dasdelen@gmail.com (M.F.D.); zuleyha.seyhan@std.medipol.edu.tr (Z.S.); omer.unlu1@std.medipol.edu.tr (O.U.); zehra.kuzu@std.medipol.edu.tr (Z.B.D.); rhoruz@medipol.edu.tr (R.H.); mehmetkocak@medipol.edu.tr (M.K.); plaguna@medipol.edu.tr (P.L.); j.j.delarosette@gmail.com (J.d.l.R.); 2 Department of Biostatistics and Medical Informatics, Istanbul Medipol University, 34810 Istanbul, Türkiye; 3 School of Medicine, Istanbul Medipol University, 34810 Istanbul, Türkiye; msargolzaeimoghaddam@std.medipol.edu.tr (M.S.); msargol@std.medipol.edu.tr (A.S.); salbayrak@medipol.edu.tr (S.A.); 4 Department of Urology, Istanbul Medipol University, 34810 Istanbul, Türkiye

**Keywords:** voiding, IPSS, urologist, uroflowmetry, asymptomatic, survey, gender

## Abstract

**Background/Objectives:** LUTS and voiding dysfunctions are prevalent in urology clinics, with uroflowmetry and IPSS as the prevailing diagnostic methods. Nevertheless, objective assessment can be constrained by age, gender, and variability in the test conditions. Portable (home) uroflowmetry addresses these limitations, allowing for more natural urinary flow recordings beyond clinic confines. This study aims to characterize spontaneous voiding patterns in healthcare professionals, exploring gender differences, variability in repeated measurements, and correlations among voiding parameters, IPSS, age, and BMI. **Methods:** This cross-sectional study was conducted during the SIU 43rd Congress in Istanbul using smart uroflow devices such as the Oruba Oruflow Uroflow Recorder, which were installed in public toilets. A total of 431 healthcare professionals participated by providing demographic information and completing the IPSS questionnaire. The data analysis included uroflowmetric parameters such as maximum flow rate (Q_max_), average flow rate (Q_ave_), and voided volume (VV), in addition to IPSS and demographic data to assess the possible associations with IPSS, age, BMI, and gender differences. **Results:** Of the participants, 76% were male and 24% female, with a higher prevalence of LUTS in women. Despite no significant gender difference in voided volume, men with lower volumes demonstrated more severe LUTS. Notably, women exhibited higher Q_max_ and Q_ave_ rates irrespective of their IPSS scores, contrasting with men whose flow rates declined with age and LUTS severity. In men, the total IPSS score was inversely associated with uroflowmetric performance, particularly impacting voiding symptoms over storage symptoms. Repeated measurements revealed noteworthy variability in Q_max_ and VV, without any influence from gender, BMI, age, or symptom severity. **Conclusions:** Our findings highlight the importance of gender-specific considerations in evaluating voiding complaints through uroflowmetry and IPSS. The significant variability observed in repeated uroflowmetry studies underlines the need for multiple measurements. Overall, this research emphasizes the significance of portable (home) uroflowmetry and calls for a reassessment of normal voiding standards in (non) clinical settings.

## 1. Introduction

Urologists are frequently consulted by both genders because of micturition complaints [1,2]. To evaluate and study the possible cause of impaired voiding, a combination of various assessment tests are considered, including uroflowmetry and the International Prostate Symptom Score (IPSS) [3,4,5].

Uroflowmetry stands out as the most frequently employed noninvasive urodynamic test to evaluate voiding performance [6]. It is routinely used to objectively assess the severity of Lower Urinary Tract Symptoms (LUTS) in men with Benign Prostatic Hyperplasia (BPH) and to monitor the response to therapy [6], and in females to evaluate Lower Urinary Tract Dysfunction (LUTD) [7,8]. Uroflowmetry is cost-effective, readily available, and demands minimal preparation time while delivering prompt results [9]. Although uroflowmetry is a simple test, factors such as voided volume, voiding position, age, psychological state, and gender might affect the micturition parameters [6,10,11,12,13].

Uroflowmetry is typically performed in an outpatient setting at designated facilities, where individuals urinate into a uroflow meter, mostly at a scheduled time. This ‘unnatural’ voiding on demand with varying bladder capacities may lead to significant test variability, suggesting the need for repeated uroflowmetry measurements and possible multiple clinic visits [9,14]. This has propelled the development of portable (home) uroflowmetry, facilitating a more natural urinary flow recording [9,15].

The International Prostate Symptom Score (IPSS) is an eight-question written screening instrument used to assess the severity of LUTS and LUTD and rapidly categorize them, aiding in the management and monitoring the response to treatment [2,5,16,17]. It effectively characterizes discomfort and its impact on the quality of life in both males and females [18], encompassing symptoms like incomplete emptying, increased frequency, urgency, slow stream, straining, and nocturia.

The present study aimed to characterize spontaneous or physiological voiding patterns and their possible variations outside of the scope of the urological consultation. Secondarily, we studied possible gender differences and scrutinized the variations in individuals’ utilization of multiple toilet visits. Finally, we explored the relationship between factors including IPSS, age, BMI, and uroflow performance.

## 2. Materials and Methods

### 2.1. Type of Study, Setting, and Participants

We conducted a cross-sectional study during the SIU 43rd Congress, held 11–14 October 2023 in Istanbul (TR). The study protocol was approved by the Ethics Committee of Istanbul Medipol University (E-10840098-772.02-6587) (2023/837).

The announcements of the study were placed at the registration area and near the exhibit area to present the voiding project. We invited symptomatic healthcare professionals attending the congress to use smart uroflow devices integrated into public toilets for males and females at the convention center (Supplementary Figure S1). This allowed participants to follow their regular routines without any imposed anxiety during urination. After explanation and upon agreement, the participant was provided with a small barcode and requested to complete an online survey including a consent form, demographic information: age, height, weight, and nationality, and a standard IPSS questionnaire with a quality-of-life question at the end (Supplementary Materials). Subsequently, participants were instructed to use the uroflowmetry devices with their barcode whenever they felt the desire to void, replicating their daily routines. Participants were encouraged to use the uroflow recorder multiple times using the same barcode throughout the congress along the 3 days of the meeting’s duration.

### 2.2. Data Collection

**Figure 1:**
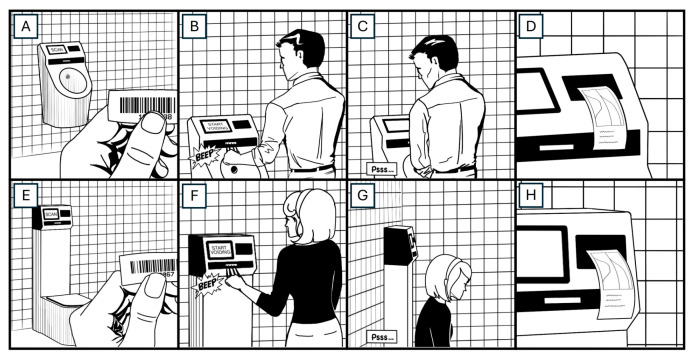
Usage of the portable uroflowmetry. Participants are provided with a unique barcode before entering the restroom (**A**,**E**). After scanning the barcode (**B**,**F**), the device initiates data recording. Participants can commence voiding within the subsequent minute (**C**,**G**). Upon completion of voiding, the device automatically prints the results out, followed by self-cleaning (**D**,**H**).

Uroflowmetry measurements were performed using a CE-certified and FDA-registered self-operating Oruba Oruflow Uroflow Recorder^®^ (Cankaya, Ankara, Türkiye. Two Oruflow-i devices were set up near the exhibit area in the men’s restroom with partitions between them to ensure privacy, and one Oruflow device was installed in a separate room for women’s use. The devices were calibrated before the initiation of the study. Upon scanning the barcode, the device would enter the recording mode. The recording began at the initiation of voiding and concluded with the cessation of voiding. The device printed the voiding curve onto thermal paper for the users’ assessment and transmitted the results via Wi-Fi to the cloud system immediately (Figure 1A–H).

### 2.3. Data Analysis

Participation was anonymous and the barcodes used for the test and calibration purposes were discarded before the analysis. Maximum urinary flow (Q_max_), average urinary flow (Q_ave_), voided volume (VV), voiding time (VT), flow time, delay time, and time to Q_max_ parameters were extracted computationally from the uroflow recordings [19]. Participants who had a voided volume of zero or exhibited invalid flow rates or had a missing information in IPPS survey were excluded from the study.

We investigated associations between voiding patterns and various parameters including age, BMI, IPSS subscores and individual IPSS components, QoL, Q_max_, Q_ave_, time to Q_max,_ and flow time in female and male subjects, stratified by voided volume and total IPSS score. We have chosen a cut-off uroflow volume of 150 mL to determine possible associations. This cut-off value is considered to distinguish between a sufficient from an insufficient voided volume [20]. Similarly, an IPSS score >7 was considered to represent ‘prominent’ symptoms and was analyzed accordingly [21].

In patients with multiple uroflows, the uroflow parameters with the highest voided volume were selected for further analysis.

In participants with >1 uroflow, repeated measurements were analyzed separately to assess variability and variance differences. Data from the participants were visualized and statistically analyzed using Python 3.10. We used median and interquartile range for categorical variables, including the total IPSS score, individual IPSS questions, and quality of life, as well as means and standard deviation for continuous variables such as age, BMI, and uroflow parameters. Statistical comparisons between groups were made using a Mann–Whitney U test. Spearman’s correlation test was applied to examine the relationships between IPSS, BMI, age, and uroflow parameters. *p* values lower than 0.05 were considered significant. Regression lines were fit between age, BMI, and uroflow parameters and reported with R2 values. Multivariate regression models have been developed to analyze the effects of independent variables such as IPSS, BMI, and age on the maximum flow rate.

## 3. Results

**Figure 2:**
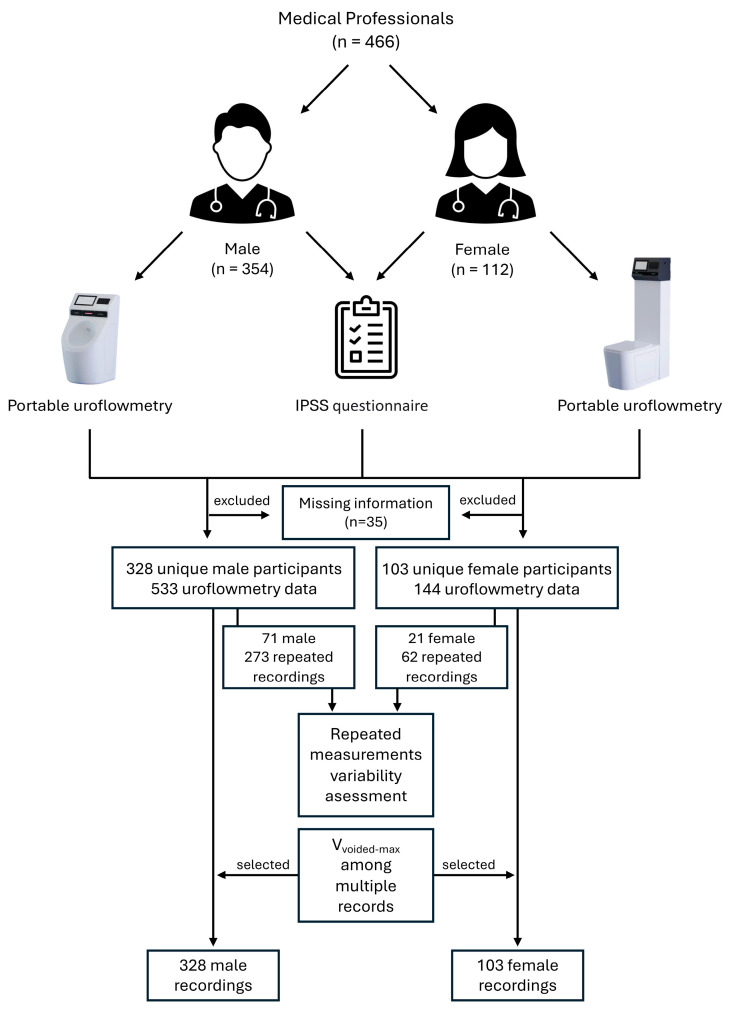
Experimental design and data analysis steps of the study.

Overall, 2252 medical professionals took part in the meeting and 466 medical professionals (~21%) volunteered to participate. Those with missing information in IPSS survey or invalid uroflowmetry recording were excluded. After removing people with missing data, we ended up with 431 participants, including 328 (76%) males and 103 (24%) females, predominantly urologists, residents, and medical students (Figure 2). By the end of the study period there were 677 uroflow recordings. The device was used multiple times by 71 males and 21 females. There were participants from 50 countries and the majority were from Türkiye (Supplementary Table S1).

Table 1 presents the baseline variables for the whole cohort and per gender. Overall, female participants were younger than males and had a lower BMI. Additionally, they were more symptomatic than males in the IPSS total score and most of the individual IPSS components. Notably, we observed that most of the complaints were related to nocturia and frequency in both genders.

### 3.1. Voided Volume

Voided volume did not differ significantly between genders in the whole cohort (Table 1). We then divided the population according to voided volume (Table 2). Males with voiding volume <150 mL were significantly older than those voiding ≥150 mL (*p* < 0.01), while age was not associated with the cut-off of 150 mL of voiding in females (*p* = 0.34). Compared to those with a voided volume ≥150 mL, men with voided volume <150 mL had higher total IPSS scores, more incomplete emptying, intermittency, weak stream, straining, and nocturia complaints, as well as lower voiding satisfaction. In women, no baseline characteristics or IPSS components were associated with a low voided volume. Expectedly, uroflow parameters such as Q_max_ and Q_ave_ were significantly lower in participants voiding <150 mL.

**Figure 3:**
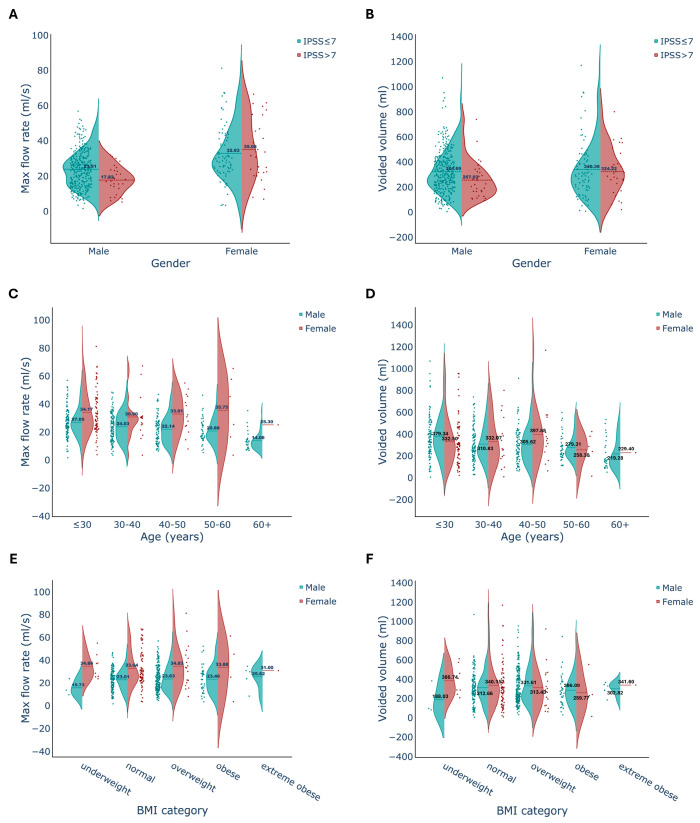
Comparison of genders, IPSS symptom groups, age groups, and BMI categories according to maximum flow rate and voided volume via violin plots. Asymptomatic-mild symptomatic (IPSS ≤7) and prominent symptomatic (IPSS > 7) groups are compared for maximum flow rate and voided volume parameters in both genders (**A**,**B**). Individuals are grouped based on age category (**C**,**D**) and BMI (**E**,**F**) and evaluated for maximum flow rate and total voided volume. BMI was categorized into 5: underweight (<18.5), normal (18.5–24.9), overweight (25–29.9), obese (30–39.9), and extreme obese (>40). The dashed lines represent the mean values.

We further evaluated whether voided volume is affected by lower urinary tract symptoms (LUTS), age, and BMI in both genders (Figure 3). We observed that in men, voided volume decreased with moderate to severe LUTS (Figure 3B) and exhibited a stepwise decline with aging (Figure 3D). However, in women, voided volume did not show a decrease with LUTS (Figure 3B), and there was no discernible pattern associated with age (Figure 3D). Additionally, BMI did not demonstrate a consistent pattern in influencing voided volume in either gender (Figure 3F).

### 3.2. Maximum Flow Rate

Females had higher maximum and average flow rates than males in general (Table 1, *p* < 0.01). The average Q_ave_ values in our study population were 12.87 mL/s for males and 17.81 mL/s for females. The average Q_max_ values were 23.35 mL/s for males and 33.51 mL/s for females. Moreover, a significantly lower time to Q_max_ and flow time were exhibited in females than in males (*p* < 0.01).

Male participants with prominent symptoms, indicated by an IPSS score higher than 7, exhibited a lower Q_max_ (Figure 3A). In contrast, female participants displayed a similar Q_max_ across different IPSS score groups. The maximum flow rate in male participants decreased progressively across age groups, while in female participants, it remained relatively consistent across different ages (Figure 3C). No significant differences were observed in the maximum flow rate among different BMI groups (Figure 3).

### 3.3. IPSS

Table 3 demonstrates the comparison of the participants according to the IPSS total score in both genders. In parallel with the previous findings, age was insignificant for females (*p* = 0.84); however, males with IPSS ≤ 7 were significantly younger than those with IPSS > 7 (*p* < 0.01). Additionally, in women, no uroflow parameters, including maximum and average flow rates, showed significant changes associated with the IPSS. In contrast, for men, while the maximum and average flow rates changed significantly with the IPSS, the time to maximum flow rate and the flow time did not show any significant variation.

### 3.4. Correlation of Uroflow Parameters

**Figure 4:**
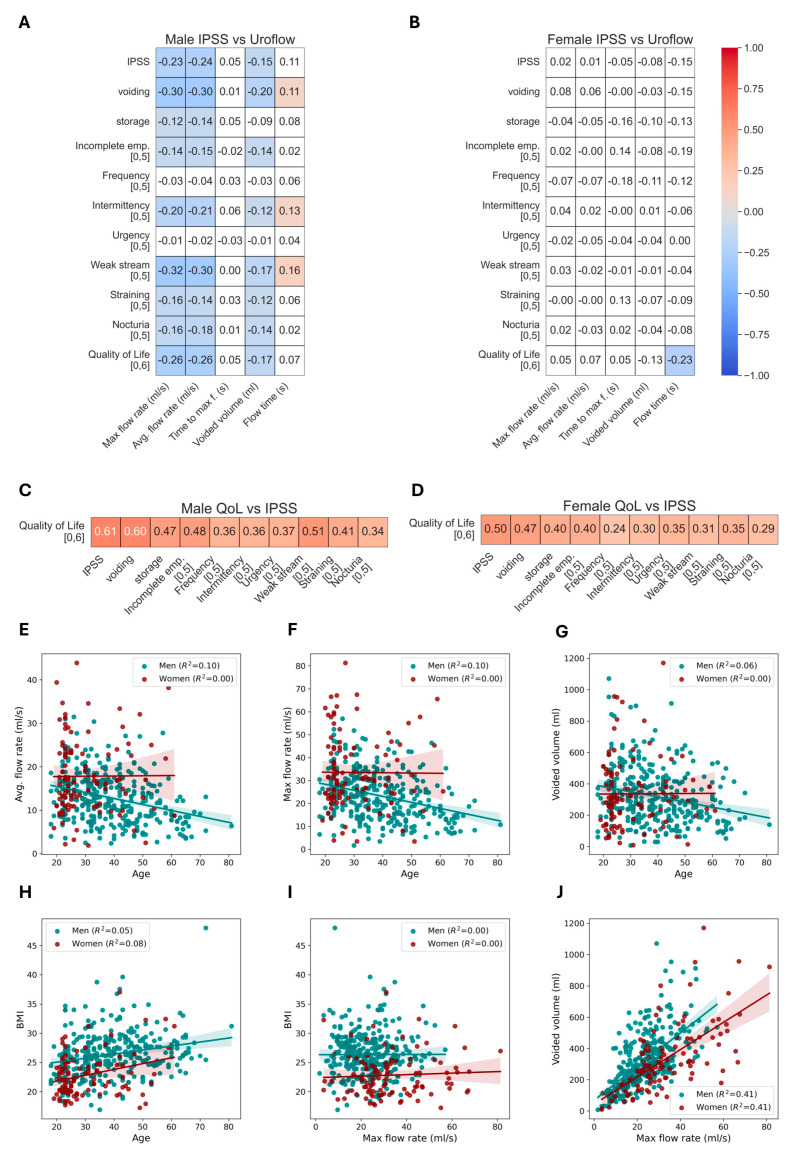
Correlation matrix identifying the correlation between the IPSS-uroflow parameters and QoL-IPSS components. For both male (**A**) and female (**B**) participants, the relationships between the components of the IPSS and uroflowmetry parameters are described separately. The color-coded heatmaps represent the strength and direction of correlation: blue signifies a negative correlation, red denotes a positive correlation, and white indicates no significant correlation. The correlation of QoL with each IPSS subscore is shown for males (**C**) and females (**D**). The strength of these correlations is visually represented through color heatmaps, where blue indicates negative correlations and red indicates positive ones. Scatter plots between continuous variables were created (**E**–**J**). Linear regression models were fit to describe their relationships. Lines show the model’s output for perfect fit between two variables. Shadows represent confidence intervals (95%).

In males, the total IPSS score, subscores, and all individual components displayed significant negative correlations with maximum flow rate and voided volume in varying degrees, suggesting a consistent pattern of increased lower urinary tract symptoms associated with decreased uroflowmetric performance (Figure 4A). The strongest negative correlation was observed between the weak stream and maximum flow rate (r = −0.32, *p* < 0.05). Voiding symptoms showed a higher degree of correlation than storage symptoms. The flow time showed a positive correlation with voiding symptoms, especially with intermittency and weak stream problems (Figure 4A). In females, the only association observed was flow time and QoL (r = −0.23, *p* < 0.05) (Figure 4B).

We also evaluated the correlation between all IPSS components and the quality of life to determine which parameter most significantly affects individuals’ satisfaction with their urinary condition. The total IPSS score showed the highest correlation with the QoL question for both males and females (*p* < 0.05, r = 0.61 and r = 0.50, respectively). Voiding symptoms had a greater impact on satisfaction regarding urinary conditions than storage symptoms. In males, the weak stream was the IPSS component most strongly correlated with quality of life (r = 0.51), while in females, incomplete emptying showed the strongest correlation (r = 0.40) (Figure 4C,D).

The difference in the age–uroflow relationship between genders can also be observed from the linear regression analysis (Figure 4E–G). The Q_max_, Q_ave,_ and voided volume show a decline over age in males but not in females. In an independent demographic plot, the BMI by age reveals similar trends and confidence intervals for both genders, with the exception of a steeper slope for females (Figure 4H). In contrast, the relationship between BMI and maximum flow rate exhibited a slope-free pattern, leading to a widened confidence interval for both genders, particularly noticeable at lower BMI values in females (Figure 4I). As expected, the voided volume and maximum flow rate showed parallel increases for both genders (Figure 4J).

Finally, we investigated whether any pre-test characteristics—such as age, gender, BMI, symptom severity, or specific symptoms—could predict outcomes in uroflowmetry parameters (Supplementary Tables S2 and S3). We found that increased age and moderate or severe LUTS were significantly associated with a decrease in Q_max_ in males. Additionally, in men, Q_max_ was notably influenced by symptoms of intermittency (OR = 0.06, *p* < 0.01), weak stream (OR = 0.04, *p* < 0.01), and straining (OR = 0.04, *p* < 0.01). However, none of these factors showed a significant association in females.

### 3.5. Repeated Measurement

**Figure 5:**
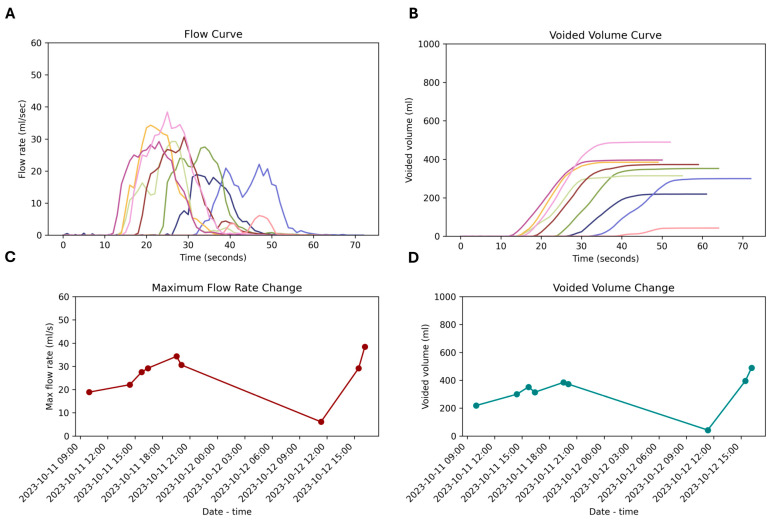
Temporal variability in uroflowmetry metrics of a single subject. Uroflowmetry data obtained from an individual at different time points were depicted. Flow curves (**A**) and voided volume curves (**B**) demonstrate noticeable variability in multiple measurements within the same individual. Each curve represents a distinct testing session, illustrating the dynamic nature of urinary flow rates and volumes. The variation in maximum flow rate (**C**) corresponds to changes in the total voided volume (**D**).

We investigated the daily voiding variability. The uroflow curves (Figure 5A) and corresponding total voided volumes (Figure 5B) of a person throughout the congress are illustrated in Figure 5. Not only the uroflow curves but also Q_max_ and total voided volume show high variability between different voidings (Figure 5C,D). The high correlation between Q_max_ and voided volume can also be observed from here. The distribution of average flow rate and voided volume values over date and time, derived from multiple measurements of 93 participants, are represented in Supplementary Figure S2. Participants with a daily voiding rhythm exhibited noticeable variations between measurements. Even with 4–9 repeated measurements, significant fluctuations, including extreme values and marked increases or decreases, were observed for both Q_max_ and voided volume.

Moreover, we investigated whether any factors could be influencing this variability among participants. Factors such as gender (Supplementary Figure S3A,B), age (Supplementary Figure S3C,D), having prominent symptoms (Supplementary Figure S3E,F), and BMI (Supplementary Figure S3G,H) were evaluated. However, none of these factors significantly affected the variances observed between different voiding events.

## 4. Discussion

Women displayed a higher Q_max_ regardless of IPSS scores, while men’s flow rates decreased with age and LUTS severity. The total IPSS score correlated negatively with uroflowmetric performance in men, with voiding symptoms showing a higher impact than storage symptoms. Repeated measurements revealed a significant variability in Q_max_ and VV, unaffected by gender, BMI, age, or symptom severity.

### 4.1. Physiological Voiding Patterns and Portable (Home) Flowmetry

In the outpatient clinical scenario, uroflowmetry potentially encounters challenges including patient indisposition due to travel, cost, time, and more importantly the psychological stress of being in a clinic. Consequently, this can eventually impact the study results [14,15]. Thus, conducting the test at home or in an out-clinic setting is seen as a more appealing alternative, since it allows for (multiple) natural flow measurements. At present, several reliable, comfortable, and telemetric home flowmeters for early diagnosis and follow-up purposes are available [14,15,22].

Here, we used Oruflow portable toilets which have integrated uroflowmetry and a self-cleaning system. The Oruflow enables the capture of the most natural voiding pattern of individuals by providing a comfortable and private environment without any stressors. To ensure privacy and minimize the potential psychological ramification on normal voiding behavior, we have installed the devices in separate rooms designated for women, as illustrated in Supplementary Figure S1. For men, we have placed barriers between the devices to enhance privacy (Supplementary Figure S1). Moreover, the design of the portable uroflowmetry devices closely resembles that of standard toilets, which helps minimize the participants’ perception of being evaluated and the pressure that their voiding is being measured. These adjustments aim to preserve the natural voiding behavior of the participants, thereby enhancing the validity of our findings. In a comparative study of self-administered and assistant-supervised uroflowmetry within a hospital setting, the use of the Oruflow is easily adapted by patients with a brief instruction and it yields statistically better data [6]. Therefore, it is anticipated that the portable out-clinic uroflowmetry conducted in this current study will yield even more optimal data.

The present study is unique in its design since we captured data on voiding information using the Oruflow device from a real-life population-based scenario in both genders without age limits and within the concept of convenience sampling. Research has demonstrated that home uroflowmetry is a promising method for both diagnosis and follow-up. This approach can be seamlessly integrated with mobile apps, enhancing its accessibility and convenience for both doctors and patients. Additionally, our findings reveal that individuals experience significant daily variations in uroflowmetry readings. Therefore, to accurately assess LUTS, we suggest conducting multiple uroflowmetry measurements under natural, daily-life conditions.

Additionally, we adopted a unique approach to evaluate voided volumes, diverging from typical clinical practices where normally, voided volumes less than 150 mL are not considered for analysis, and volumes greater than 150 mL are recommended for detailed examination. Our methodology focused on replicating everyday routines by allowing participants to void naturally, whenever they felt the need. This approach was aimed at capturing a more realistic picture of voiding patterns as they occur in daily life, rather than under controlled clinical conditions [6]. By including all participants regardless of their voided volume, we gained a broader understanding of voiding behavior across a diverse population. A total of 21 females and 48 males voided below 150 mL as their maximum voided volume throughout the study. In a comprehensive uroflowmetry study searching whether voided volume <150 mL is an unreliable test result or a sign of severe storage symptoms, it was shown that 50% of men with storage symptoms cannot achieve 150 mL voided volume on initial uroflowmetry, and suggested that the clinicians should consider the voided volume on initial uroflowmetry to predict the severity of storage symptoms [11]. Additionally, bladder outlet obstruction (BOO) is seen more frequently in those voiding less than 150 mL regardless of their maximum flow rate [23]. Therefore, we believe in the importance of identifying the characteristics of those people. They were older and more symptomatic compared to those who voided more than 150 mL in males, which is consistent with previous publications [11]. Specifically, they reported having more nocturia and intermittency problems as well as less urinary condition satisfaction. Expectedly, their voiding performance was lower in males. However, it is noteworthy that this study was conducted in a population-based environment, not in a hospital setting. As a result, most participants are considered asymptomatic. This study elegantly demonstrates that what is often classified as abnormal, such as voiding less than 150 mL, might actually be a part of a normal voiding routine.

### 4.2. Gender Differences

The present study enabled us to thoroughly examine voiding patterns from both objective and subjective perspectives. For the subjective analysis of voiding, we utilized the IPSS. IPSS was originally developed to evaluate male voiding issues, particularly those associated with Benign Prostatic Hyperplasia (BPH) [21]. Yet, it has gained broader application. In the current literature, the most comprehensive population-based studies supported the validity, reliability, and sensitivity of the IPSS for assessing females with lower urinary tract symptoms [5,24]. In another population-based study, even age and gender-stratified normative values for the IPSS for older community-living females were identified [25]. Despite some influence from subject backgrounds, psychometric analysis revealed that the IPSS remains relevant for examining women as well as men [26]. Additionally, a Korean population-based study highlighted the usefulness of storage symptom scores in IPSS for evaluating female LUTS in Korean women (Lee D H, ICS 2013-Abstract 810). Even though there are alternative scoring systems supported by different communities, IPSS seems the most commonly used and reliably validated index in the common urology practice and the existing literature [24]. We used a cut of value of 7 to classify symptom severity. In this study, it was observed that females had higher values in the IPSS total score and most of the individual IPSS components than males. In parallel with our findings, a population-based study of men and women showed that the incidence of moderate to severe LUTS is significantly higher for women according to the AUASI scale [27]. Although the incidence increased with age, it had a plateau among women between ages 50 and 70 years of age and then doubled to 35.0% among women ≥70 years. These findings supported the more dynamic nature of women’s symptoms across the age span that may indicate fluctuations in underlying contributors, such as recent reproductive experiences (e.g., childbirth) or the hormonal milieu (e.g., menopausal transition), and may be more related to an overactive bladder, rather than voiding obstruction. In contrast, the LUTS in men often reflected the development of benign prostatic obstruction or hyperplasia, which is generally slow and protracted [27]. However, a cohort study, aiming to explore age and gender stratified normative IPSS values for adults aged 60 years and older, displayed higher scores for men in the IPSS total and individual components except for the 70–74 years age group [25]. Additionally, a notable increase with a more linear trend was revealed in males. In conclusion, they proposed that men are more willing to report more symptoms than women for the majority of the IPSS questions among the older age groups [25]. In fact, one of the largest population-based survey studies solved the mystery beyond the gender-specific IPSS score issue: it was discovered that IPSS and individual component scores are higher in women younger than 49 years of age and balanced around 50–59 years of age [28]. Conversely, men have higher scores after 60 years of age. Eventually, they reached a close agreement showing the lack of gender specificity of IPSS [28].

For the objective analysis of voiding, we performed a uroflowmetry analysis. Women in the same age groups exhibited significantly higher maximum flow rates (33.51 mL/s) and average flow rates (17.81 mL/s) compared to men, who showed rates of 23.35 mL/s and 12.87 mL/s, respectively. This observation is consistent with existing literature [29] and the difference is commonly attributed to the anatomical distinctions between the genders, such as females having a shorter urethra and the absence of a prostate gland. The mean average and maximum flow rate values are highly variable among age groups and different populations. Previous studies reported varying maximum flow rates in healthy males: 17.4 mL/s in Brazilian population [30], 22.8 mL/s in Indian population [31], 28.4 mL/s in Australian adolescents [32], and 27.8 mL/s in Thai subjects [29]. A systematic review of healthy women reported pooled estimates of 29 mL/s (range 26–32 mL/s) for Q_max_ and 15 mL/s (range 12–18 mL/s) for Q_ave_ [33]. The values for males in our study appear to be an average of the literature, while our female population is at the higher limit. This discrepancy between our findings and those of other studies can be attributed to the differing characteristics of the participants.

We observed lower Q_max_, Q_ave,_ and VV values in males IPSS > 7 compared to IPSS ≤ 7, while females did not show such a difference in our study. Regarding the IPSS and uroflowmetric parameters, our study established the correlations among males; however, females did not display any correlation except between the quality of life and flow time. Although several previous studies explored various correlations between the IPSS total score, subscores, IPSS V/S ratio, and different uroflowmetric parameters [3,4], a prospective multi-center study analyzing female voiding dysfunction found no statistically significant difference in IPSS scores and subscores for different cutoff values of maximum flow rate, which is used to grade female voiding difficulty [10]. Consistent with our findings, an increase in IPSS scores across consecutive age groups without associated complaints was demonstrated in a cross-sectional study of Dutch adults reporting no voiding complaints [34]. They suggested that voiding symptoms, even in the moderate to severe range, may not be perceived as complaints, potentially due to being considered as a part of the natural aging process. On the other hand, the younger age mean in our study population might contribute to the lack of correlation in females compared to other studies.

Although the IPSS is validated for use in both genders, it is important to recognize that it captures different domains in men and women. While it effectively complements and accurately reflects the objective performance in males, it seems to be less representative of female voiding issues. A gender-specific scoring system may be more suitable for assessing female voiding problems.

### 4.3. Multiple Voiding Visits

This study also explored the variability in voiding patterns, analyzing multiple measurements from participants. In parallel with our findings, a wide variation within repeated measurements was stated for both genders in several studies consisting of small healthy populations [7,12]. In another study assessing intra-individual variability through office-based uroflowmetry, no statistically significant variability was observed in healthy women and the findings suggested that a single uroflow measurement could reliably represent the patient’s voiding pattern [35]. Conversely, in the current study, significant fluctuations were noted in both maximum flow rate and voided volume across multiple measurements, regardless of gender, BMI, or symptom severity. Therefore, these findings and the current literature review underscore the complexity of voiding patterns and their multifactorial influences, highlighting the importance of considering a range of demographic and symptomatic factors in urological assessments and treatments.

### 4.4. Relationships between Uroflow and Participants’ Characteristics

Consistent with previous research, our study also observed that the voiding performance in males tends to decrease with age [31,36,37]. The absolute decrease in average flow rate and max flow rate per life decade was 1.39 mL/s and 2.62 mL/s, respectively. However, the age-flow rate relation in females is relatively controversial. Some studies suggest that there is no significant dependence of urinary flow rates for women [8,37,38,39], while some have shown the declining effect of age on uroflow performance [31,36,40]. This lack of consensus may be due to varying characteristics of the study groups. For instance, research by Stephan et al. focused on patients over 40 years old, highlighting that bladder capacity and voided volume in females actually begin to decline after 60 years of age [36]. Another community-based study showed a significant difference only between premenopausal and postmenopausal women [31]. Considering that females do not have an age-dependent enlarging prostate and the reduction in Q_max_ and Q_ave_ is likely due to a decrease in voided volume, we did not observe an age-dependent decline in voiding performance in females under 60 years of age. However, a multivariate study claims the presence of an independent variable on age-flow rate relation in females, although it is not clearly defined. Our findings for females <60 years old are mostly consistent with other studies, but the younger and asymptomatic cohort of our study may obscure the true effect of age on urinary flow rates and our findings cannot be generalized for females over 60 years old.

The quality of life (QoL) correlations further emphasized the impact of voiding symptoms, with the total IPSS score showing the highest correlation with QoL questions in both males and females. Similar strong correlations within various populations were shown in the current literature [2,17]. The data indicated that voiding symptoms had a more substantial impact on urinary condition satisfaction than storage symptoms, with ‘weak stream’ in males and ‘incomplete emptying’ in females being the most impactful symptoms. These findings are consistent with the existing literature for females [1]; however, the most impactful item for males is showing variations, according to different studies, most probably showing the effect of the study population [5,41].

Further analysis revealed that body mass index (BMI) did not significantly influence voided volumes or flow rates in either gender. However, symptom severity, as measured by the IPSS, showed a clear impact: males with more severe symptoms (IPSS > 7) had lower flow rates and voided volumes, whereas in females, flow rates and voided volumes were similar across different IPSS score groups. Additionally, our study highlighted the significant negative correlation between total IPSS score and uroflowmetric parameters like maximum and average flow rates in males, suggesting that increased LUTS are associated with decreased uroflowmetric performance. Consistent with the findings, several studies showed negative correlations between IPSS and various uroflowmetric parameters in males [16,30,42]. In contrast, the IPSS-uroflowmetric parameter relation in females has not been studied well in the literature.

Considering the gender differences in uroflowmetry results, this method is critical for diagnosing male voiding symptoms. However, for female voiding issues, it may not be as sufficient, since uroflow parameters typically remain consistent until the age of 60 and do not exhibit variations with the severity of LUTS as they do in males.

### 4.5. Limitations

One of the limitations of our study is the lack of information regarding whether participants were undergoing any medical treatment specifically for their voiding issues or if they had undergone any surgical interventions, such as the Transurethral Resection of the Prostate (TURP). This could potentially influence the study’s findings. Additionally, our knowledge about the participants’ comorbid conditions, such as diabetes mellitus, or their use of certain medications, like antihypertensive drugs, is limited. These factors could have a significant impact on voiding patterns and urinary function, thus affecting the results. Another constraint of our study is the relatively small sample size of female participants. This disparity in gender representation might limit the generalizability of our findings across both genders, potentially leading to less comprehensive insights into the voiding patterns of female participants compared to their male counterparts. Although participants were told to void whenever they needed without pressure and uroflowmetry devices were embedded inside the toilets, we do not know whether participants were stressed due to their participation in this study as it was not captured in the survey. Also, it might be necessary to compare study flowmetry results that are captured at the meeting and an in-clinic flowmetry result of the same persons.

## 5. Conclusions

In a clinical setting, a voided volume of less than 150 mL is deemed ‘non-representative’ for effectively evaluating voiding complaints. Notably, a considerable proportion of participants exhibited a voided volume under this threshold. Among these individuals, men displayed higher total IPSS scores and more pronounced symptoms of incomplete emptying, intermittency, weak stream, straining, and nocturia, along with a lower satisfaction in voiding. Conversely, in women, no baseline characteristics or components of the IPSS were linked to the low voided volume. Consequently, these symptomatic (or asymptomatic) participants often showed ‘prominent’ symptoms.

Remarkably, uroflowmetry revealed similar Q_max_ values for women regardless of their IPSS, unlike men who had significantly decreased Q_max_ in the prominent symptomatic group. In males, Q_max_ progressively decreased with age, while no such association was found in females.

Finally, repeated uroflowmetry is a common clinical practice to reliably record representative voiding parameters. Interestingly, repeated measurements revealed a significant variability in maximum flow rates and voided volume, irrespective of gender, BMI, age, or symptom severity. These observations suggest the need to reassess what constitutes normal voiding standards in both clinical and non-clinical settings.

## Figures and Tables

**Table 1:** Characteristics and comparison of the study populations. Female and male participants are presented separately.

Parameters	Whole Cohort (*n* = 431)	Female (*n* = 103)	Male (*n* = 328)	*p* Values
Age (years)	36.87 ± 12.77	30.20 ± 10.33	38.96 ± 12.76	<0.01
BMI (kg/m^2^)	25.51 ± 4.04	22.82 ± 3.70	26.35 ± 3.77	<0.01
IPSS Total Score	2.0 (5.0)	4.0 (6.5)	2.0 (4.0)	<0.01
Incomplete Emptying	0.0 (0.0)	0.0 (1.0)	0.0 (0.0)	<0.01
Frequency	0.0 (1.0)	1.0 (2.0)	0.0 (1.0)	<0.01
Intermittency	0.0 (0.0)	0.0 (1.0)	0.0 (0.0)	<0.01
Urgency	0.0 (0.0)	0.0 (1.0)	0.0 (0.0)	<0.01
Weak Stream	0.0 (1.0)	0.0 (1.0)	0.0 (0.25)	0.23
Straining	0.0 (0.0)	0.0 (0.0)	0.0 (0.0)	0.17
Nocturia	0.0 (1.0)	1.0 (1.0)	0.0 (1.0)	0.03
QoL	0.0 (1.0)	1.0 (2.0)	0.0 (1.0)	<0.01
Qmax (mL/s)	25.78 ± 12.70	33.51 ± 16.05	23.35 ± 10.34	<0.01
Qave (mL/s)	14.05 ± 6.78	17.81 ± 8.62	12.87 ± 5.61	<0.01
Voided Volume (mL)	322.68 ± 184.66	336.00 ± 219.85	318.50 ± 172.31	0.40
Time to Qmax (s)	8.88 ± 4.83	6.78 ± 3.93	9.54 ± 4.90	<0.01
Flow Time (s)	23.79 ± 10.95	19.17 ± 10.36	25.24 ± 10.74	<0.01

**Table 2:** Characteristics and comparison of the study populations. Female and male participants are presented separately according to the voided volume.

Parameters	Female (*n* = 103)			Male (*n* = 328)		
	V_voided_ < 150 (*n* = 21)	V_voided_ ≥ 150 (*n* = 82)	*p* Value	V_voided_ < 150 (*n* = 48)	V_voided_ ≥ 150 (*n* = 280)	*p* Value
Age (years)	28.33 ± 9.14	30.68 ± 10.61	0.35	43.62 ± 14.67	38.16 ± 12.26	<0.01
BMI (kg/m^2^)	22.56 ± 3.74	22.89 ± 3.71	0.72	25.88 ± 3.99	26.43 ± 3.73	0.35
IPSS Total Score	4.0 (4.0)	4.0 (7.0)	0.66	2.0 (4.0)	1.0 (4.0)	0.03
Incomplete Emptying	0.0 (1.0)	0.0 (1.0)	0.57	0.0 (0.25)	0.0 (0.0)	0.12
Frequency	1.0 (1.0)	1.0 (2.0)	0.16	0.0 (1.0)	0.0 (1.0)	0.96
Intermittency	0.0 (1.0)	0.0 (1.0)	0.82	0.0 (1.0)	0.0 (0.0)	0.04
Urgency	0.0 (1.0)	0.0 (1.0)	0.56	0.0 (0.0)	0.0 (0.0)	0.55
Weak Stream	0.0 (1.0)	0.0 (1.0)	0.78	0.0 (1.0)	0.0 (0.0)	0.05
Straining	0.0 (0.0)	0.0 (0.0)	0.47	0.0 (0.25)	0.0 (0.0)	0.06
Nocturia	1.0 (1.0)	1.0 (1.0)	0.74	1.0 (1.0)	0.0 (1.0)	0.02
QoL	1.0 (2.0)	1.0 (2.0)	0.82	1.0 (1.25)	0.0 (1.0)	0.01
Qmax (mL/s)	17.35 ± 9.15	37.65 ± 14.78	<0.01	10.42 ± 4.27	25.57 ± 9.41	<0.01
Qavg (mL/s)	8.68 ± 3.96	20.15 ± 7.91	<0.01	5.69 ± 2.07	14.10 ± 5.07	<0.01
Voided Volume (mL)	89.88 ± 41.08	399.02 ± 201.81	<0.01	95.55 ± 32.50	356.72 ± 156.85	<0.01
Time to Qmax (s)	4.71 ± 1.74	7.30 ± 4.16	<0.01	6.96 ± 3.80	9.98 ± 4.93	<0.01
Flow Time (s)	11.62 ± 9.51	21.10 ± 9.71	<0.01	17.98 ± 7.91	26.49 ± 10.67	<0.01

**Table 3:** Characteristics and comparison of the study populations. Female and male participants are presented separately according to the IPSS.

Parameters	Female (*n* = 103)			Male (*n* = 431)		
	IPSS ≤ 7 (*n* = 75)	IPSS > 7 (*n* = 28)	*p* Value	IPSS ≤ 7 (*n* = 298)	IPSS > 7 (*n* = 30)	*p* Value
Age (years)	30.08 ± 10.04	30.54 ± 11.23	0.84	38.25 ± 12.23	46.03 ± 15.76	<0.01
BMI (kg/m^2^)	22.72 ± 3.72	23.10 ± 3.68	0.64	26.17 ± 3.74	28.10 ± 3.64	<0.01
IPSS Total Score	2.0 (3.5)	10.5 (4.25)	<0.01	1.0 (3.0)	10.5 (6.0)	<0.01
Incomplete Emptying	0.0 (0.0)	1.0 (2.0)	<0.01	0.0 (0.0)	2.0 (2.75)	<0.01
Frequency	0.0 (1.0)	2.0 (2.25)	<0.01	0.0 (1.0)	2.0 (2.0)	<0.01
Intermittency	0.0 (1.0)	2.0 (2.0)	<0.01	0.0 (0.0)	1.0 (1.0)	<0.01
Urgency	0.0 (0.0)	1.0 (3.0)	<0.01	0.0 (0.0)	2.0 (1.0)	<0.01
Weak Stream	0.0 (0.0)	1.0 (3.0)	<0.01	0.0 (0.0)	2.0 (2.75)	<0.01
Straining	0.0 (0.0)	1.0 (1.0)	<0.01	0.0 (0.0)	1.0 (1.75)	<0.01
Nocturia	0.0 (1.0)	1.0 (2.0)	<0.01	0.0 (1.0)	1.0 (1.0)	<0.01
QoL	1.0 (1.0)	3.0 (4.0)	<0.01	0.0 (1.0)	3.0 (1.0)	<0.01
Qmax (mL/s)	32.93 ± 15.58	35.08 ± 17.45	0.55	23.91 ± 10.42	17.82 ± 7.65	<0.01
Qavg (mL/s)	17.41 ± 7.88	18.87 ± 10.44	0.45	13.18 ± 5.63	9.78 ± 4.34	<0.01
Voided Volume (mL)	340.39 ± 232.46	324.22 ± 185.23	0.74	324.69 ± 173.87	257.03 ± 144.48	0.04
Time to Qmax (s)	6.53 ± 3.78	7.43 ± 4.32	0.31	9.58 ± 5.02	9.13 ± 3.55	0.64
Flow Time (s)	19.29 ± 9.71	18.82 ± 12.12	0.84	25.04 ± 10.87	27.27 ± 9.22	0.28

## Data Availability

The data presented in this study are available from the corresponding author on receipt of reasonable request and are not publicly available.

## Supplementary Materials

The following supporting information can be downloaded at: https://www.mdpi.com/article/10.3390/jcm13102857/s1, Figure S1: The pictures of Oruflow-I (left) and Oruflow-h (right). Figure S2: The distribution of average flow rate and voided volume values over multiple measurements for each gender (A–D). Figure S3: Statistical analysis of intra-individual variability between repeated measurements. The association with gender (A,B), age (C,D), symptom severity (E,F) and BMI (G,H) were evaluated. Table S1: Nationality distribution of participants. Table S2: Multivariate linear regression for maximum flow rate. Table S3: Multivariate linear regression for maximum flow rate.
