# Supplementary material for: Reassessing Normal Voiding Standards: A Cross-Sectional Study Based on Medical Professionals’ Evaluations with Portable Uroflowmetry and IPSS
Source: J Clin Med. 2024 May 12;13(10):2857. doi: 10.3390/jcm13102857 (PMC11122113; doi:10.3390/jcm13102857)
Supplement: Supplementary file 1 [file jcm-13-02857-s001.zip › jcm-2969979-supplementary.pdf]

Reassessing Normal Voiding Standards: A Cross-Sectional Study Based on Medical Professionals' Evaluations with Portable Uroflowmetry and IPSS

Supplementary File

Table of Contents

BASIC CHARACTERISTICS AND IPSS SURVEY **3**

SUPPLEMENTARY FIGURES **5**

SUPPLEMENTARY FIGURE S1 5
SUPPLEMENTARY FIGURE S2. 6
SUPPLEMENTARY FIGURES 3 7

SUPPLEMENTARY TABLES **8**

SUPPLEMENTARY TABLE S1 9
SUPPLEMENTARY TABLE S2 11
SUPPLEMENTARY TABLE S3 12

Basic Characteristics and IPSS Survey

Characteristic:

1. **Please enter your barcode:**
2. **What is your gender:**
   - Female
   - Male
   - Other (Please specify)
3. **What is your age:**
4. **What is your weight? (kg)**
5. **What is your height? (cm)**
6. **What is your nationality?**

IPSS Questionnaire:

7. **Incomplete Emptying:** Over the past month, how often have you had a sensation of not emptying your bladder completely after you finish urinating?
   (0) Not At All
   (1) Less Than 1 Time in 5
   (2) Less Than Half of The Time
   (3) About Half The Time
   (4) More Than Half The Time
   (5) Almost Always

8. **Frequency:** Over the past month, how often have you had to urinate again less than two hours after you finished urinating?
   (0) Not At All
   (1) Less Than 1 Time in 5
   (2) Less Than Half of The Time
   (3) About Half The Time
   (4) More Than Half The Time
   (5) Almost Always

9. **Intermittency:** Over the past month, how often have you found you stopped and started again several times when you urinated?
   (0) Not At All
   (1) Less Than 1 Time in 5
   (2) Less Than Half of The Time
   (3) About Half The Time
   (4) More Than Half The Time
   (5) Almost Always

10. **Urgency:** Over the last month, how difficult have you found it to postpone urination?
    (0) Not At All
    (1) Less Than 1 Time in 5
    (2) Less Than Half of The Time
    (3) About Half The Time
    (4) More Than Half The Time
    (5) Almost Always

11. **Weak Stream:** Over the past month, how often have you had a weak urinary stream?
    (0) Not At All
    (1) Less Than 1 Time in 5
    (2) Less Than Half of The Time
    (3) About Half The Time
    (4) More Than Half The Time
    (5) Almost Always

12. **Straining:** Over the past month, how often have you had to push or strain to begin urination?
    (0) Not At All
    (1) Less Than 1 Time in 5
    (2) Less Than Half of The Time
    (3) About Half The Time
    (4) More Than Half The Time
    (5) Almost Always

13. **Nocturia:** Over the past month, how many times did you most typically get up to urinate from the time you went to bed until the time you got up in the morning?
    (0) Not At All
    (1) Less Than 1 Time in 5
    (2) Less Than Half of The Time
    (3) About Half The Time
    (4) More Than Half The Time
    (5) Almost Always

14. **Quality of life due to urinary symptoms:** If you were to spend the rest of your life with your urinary condition the way it is now, how would you feel about that?
    (0) Delighted
    (1) Pleased
    (2) Mostly satisfied
    (3) Mixed
    (4) Mostly unhappy
    (5) Unhappy
    (6) Terrible

Supplementary Figures

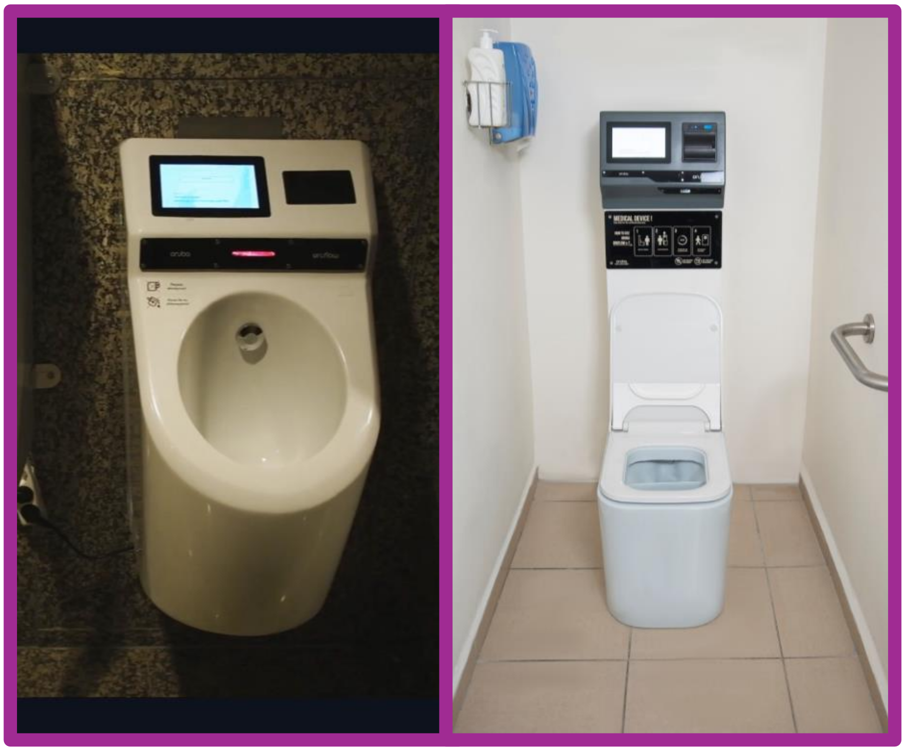

Supplementary Figure S1. The pictures of Oruflow-l (left) and Oruflow-h (right).

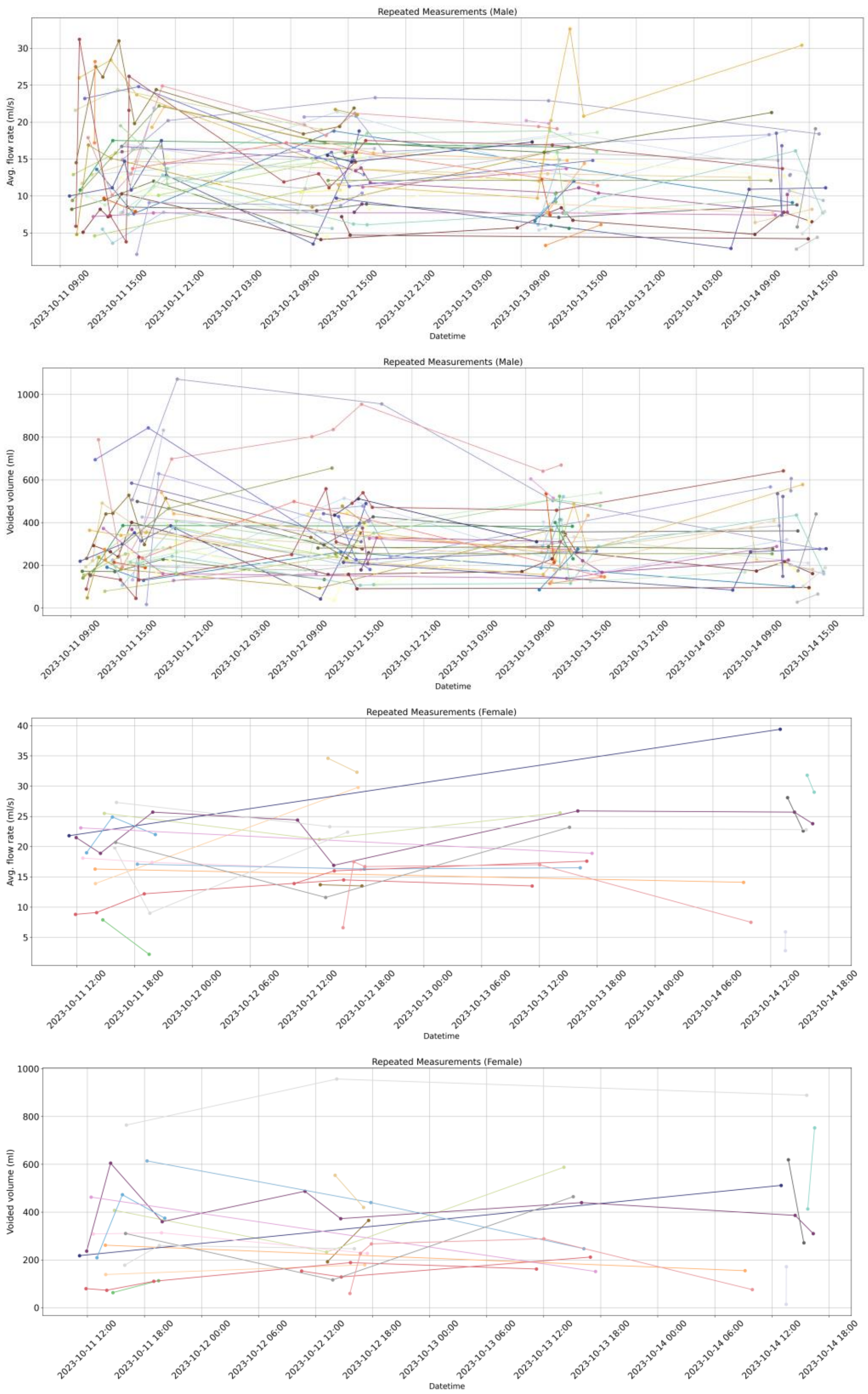

Supplementary Figure S2. The distribution of average flow rate and voided volume values over multiple measurements for each gender (A-D).

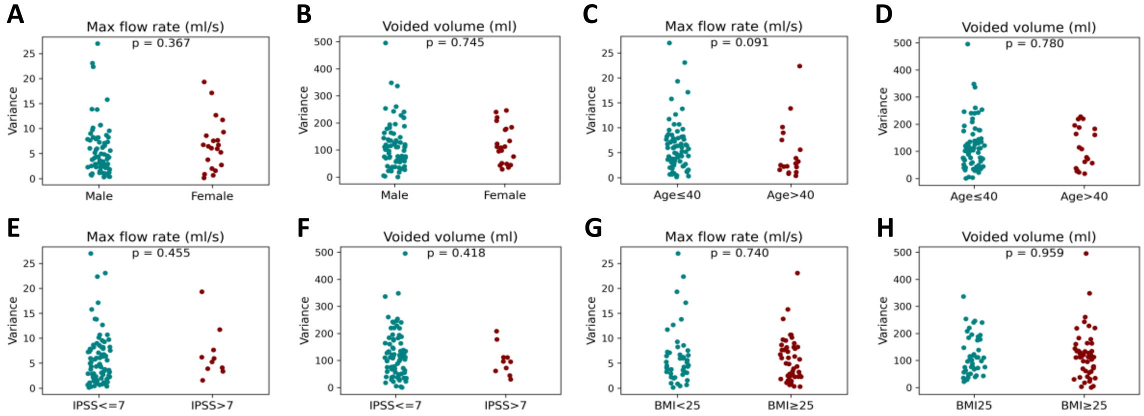

Supplementary Figure 3. Statistical analysis of intra-individual variability between repeated measurements. The association with gender (A, B), age (C, D), symptom severity (E, F) and BMI (G, H) were evaluated.

Supplementary Tables

Nationality	Count		Nationality	Count	
	Male	Female		Male	Female
**Turkish**	201	71	**Singaporean**	1	0
**Indian**	22	0	**Tanzanian**	1	0
**British**	14	1	**Serbian**	1	0
**Russian**	6	0	**Senegalese**	1	1
**Kenyan**	6	0	**Romanian**	1	0
**Canadian**	5	0	**Portuguese**	1	0
**Chinese**	5	3	**Yemenite**	1	0
**Pakistani**	5	0	**Palestinian**	1	0
**Lebanese**	4	0	**Kazakhstani**	1	1
**Australian**	4	0	**Lithuanian**	1	1
**Spanish**	4	1	**Kuwaiti**	1	0
**South Korean**	4	0	**Jordanian**	1	0
**American**	4	3	**Japanese**	1	0
**Mexican**	3	0	**Iraqi**	1	0
**Italian**	3	1	**Greek**	1	0
**Iranian**	2	3	**French**	1	2
**Indonesian**	2	0	**Egyptian**	1	0
**German**	2	4	**Dutch**	1	0
**South African**	2	2	**Bangladeshi**	1	0

**Chilean**	2	0	**Zimbabwean**	1	0
**Cameroonian**	2	0	**Nepalese**	0	1
**Belgian**	2	2	**Armenian**	0	1
**Nigerian**	2	1	**Gabonese**	0	1
**Sri Lankan**	1	0	**Finnish**	0	1
**Swiss**	1	0	**Filipino**	0	2

Supplementary Table S1. Nationality distribution of participants

Parameters	Female (n=103)			Male (n=328)		
	Coefficient	95%CI	p value	Coefficient	95%CI	p value
**Age**						
• <30	Reference	Reference	Reference	Reference	Reference	Reference
• 30-40	-3.03	[-12.47,6.41]	0.53	-3.58	[-6.35,-0.81]	0.01
• 40-50	-1.40	[-11.56,8.76]	0.79	-6.06	[-9.16,-2.97]	<0.01
• 50-60	1.23	[-15.04,17.50]	0.88	-6.07	[-9.79,-2.35]	<0.01
• 60+	-13.31	[-53.36,26.73]	0.51	-13.53	[-17.96,-9.10]	<0.01
**BMI**						
• Underweight	1.17	[-11.35,13.69]	0.85	-8.14	[-19.30,3.02]	0.15
• Normal	Reference	Reference	Reference	Reference	Reference	Reference
• Overweight	1.47	[-7.29,10.23]	0.74	2.64	[0.27,5.01]	0.03
• Obese	3.00	[-19.32,25.31]	0.79	2.73	[-1.06,6.51]	0.16
• Extreme Obese	-0.69	[-35.26,33.89]	0.97	7.42	[-1.43,16.27]	0.10
**Total IPSS**						
• ≤ 7	Reference	Reference	Reference	Reference	Reference	Reference

• > 7	2.53	[-5.17,10.24]	0.52	-5.00	[-8.77,-1.22]	<0.01

Supplementary Table S2. Multivariate linear regression for maximum flow rate

Parameters	Female (n=103)			Male (n=328)		
	Coefficient	95%CI	p value	Coefficient	95%CI	p value
**Total IPSS**	0.05	[-0.67,0.77]	0.88	-0.48	[-0.78,-0.18]	<0.01
**Incomplete Emptying**	1.48	[-1.37,4.32]	0.31	-1.19	[-2.44,0.07]	0.06
**Frequency**	-0.96	[-3.50,1.57]	0.45	-0.35	[-1.44,0.73]	0.52
**Intermittency**	0.46	[-2.30,3.22]	0.74	-2.89	[-4.33,-1.45]	<0.01
**Urgency**	-1.45	[-4.32,1.41]	0.32	-0.09	[-1.62,1.44]	0.91
**Weak Stream**	-0.58	[-4.25,3.10]	0.76	-3.11	[-4.32,-1.89]	<0.01
**Straining**	1.12	[-3.30,5.54]	0.62	-3.24	[-5.41,-1.08]	<0.01
**Nocturia**	1.44	[-1.44,4.33]	0.32	-0.21	[-1.38,0.96]	0.72
**QoL**	0.52	[-1.43,2.47]	0.60	-1.56	[-2.49,-0.64]	<0.01

Supplementary Table S3**.** Multivariate linear regression for maximum flow rate. *Adjusted for age and BMI
